# Supplementary figures and images for: Predicting maintenance lithium response for bipolar disorder from electronic health records—a retrospective study
Source: PeerJ. 2024 Oct 14;12:e17841. doi: 10.7717/peerj.17841 (PMC11485101; doi:10.7717/peerj.17841)

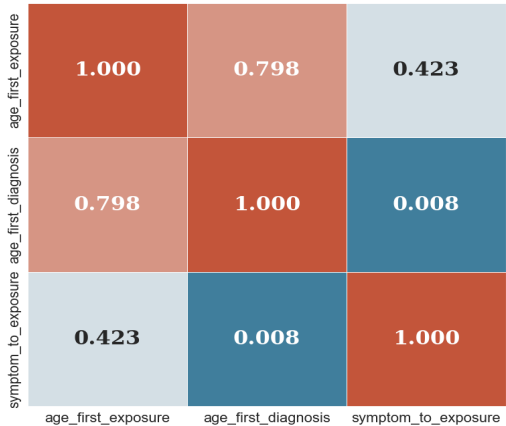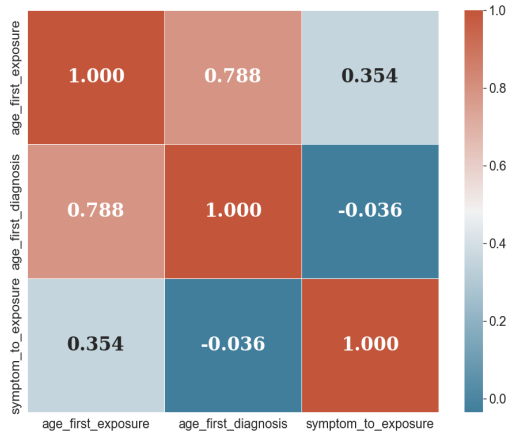

Supplement: Supplemental Information 1 [file peerj-12-17841-s001.pdf]

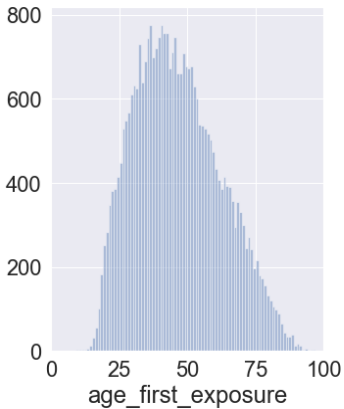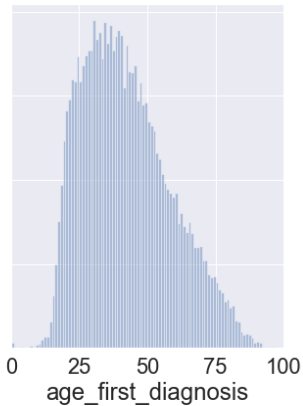

Supplement: Supplemental Information 3 [file peerj-12-17841-s003.pdf]

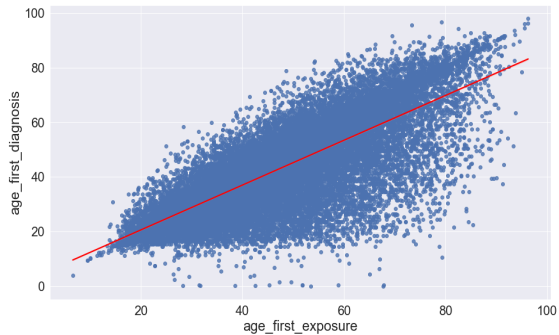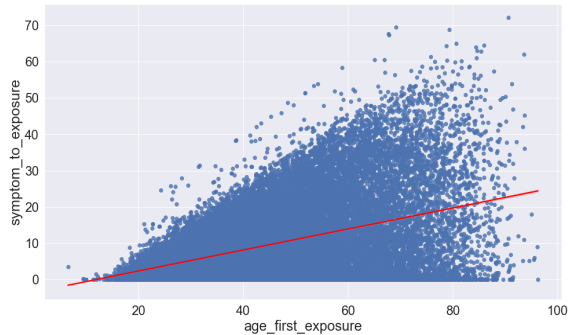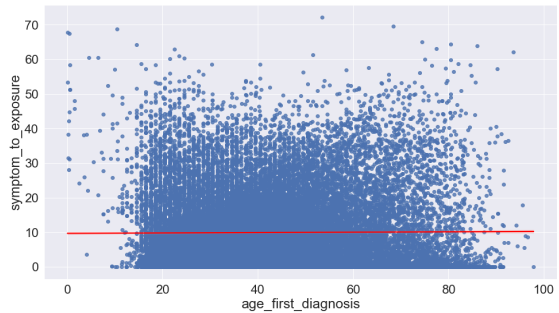

Supplement: Supplemental Information 4 [file peerj-12-17841-s004.pdf]

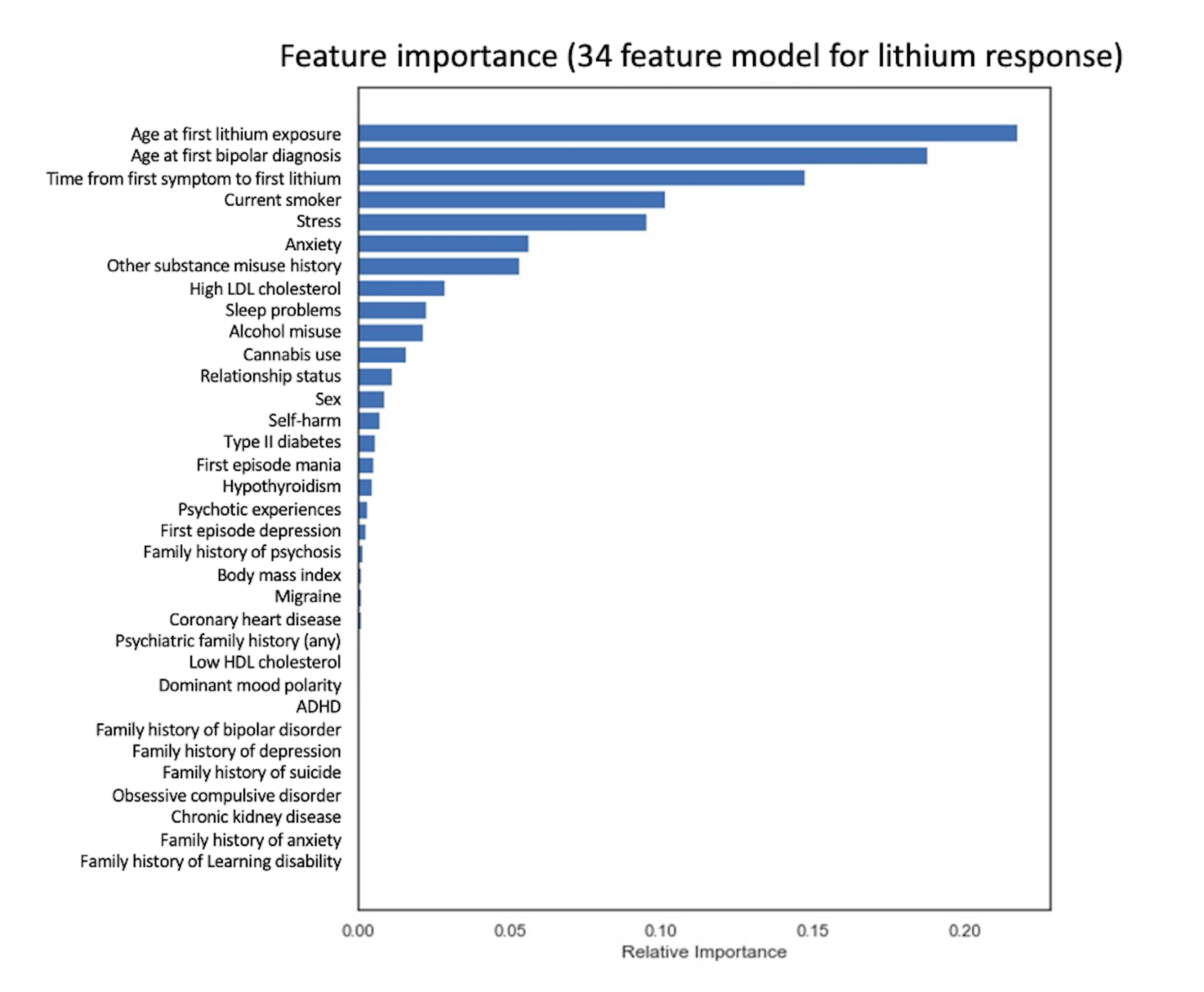

Supplement: Supplemental Information 5 [file peerj-12-17841-s005.png]

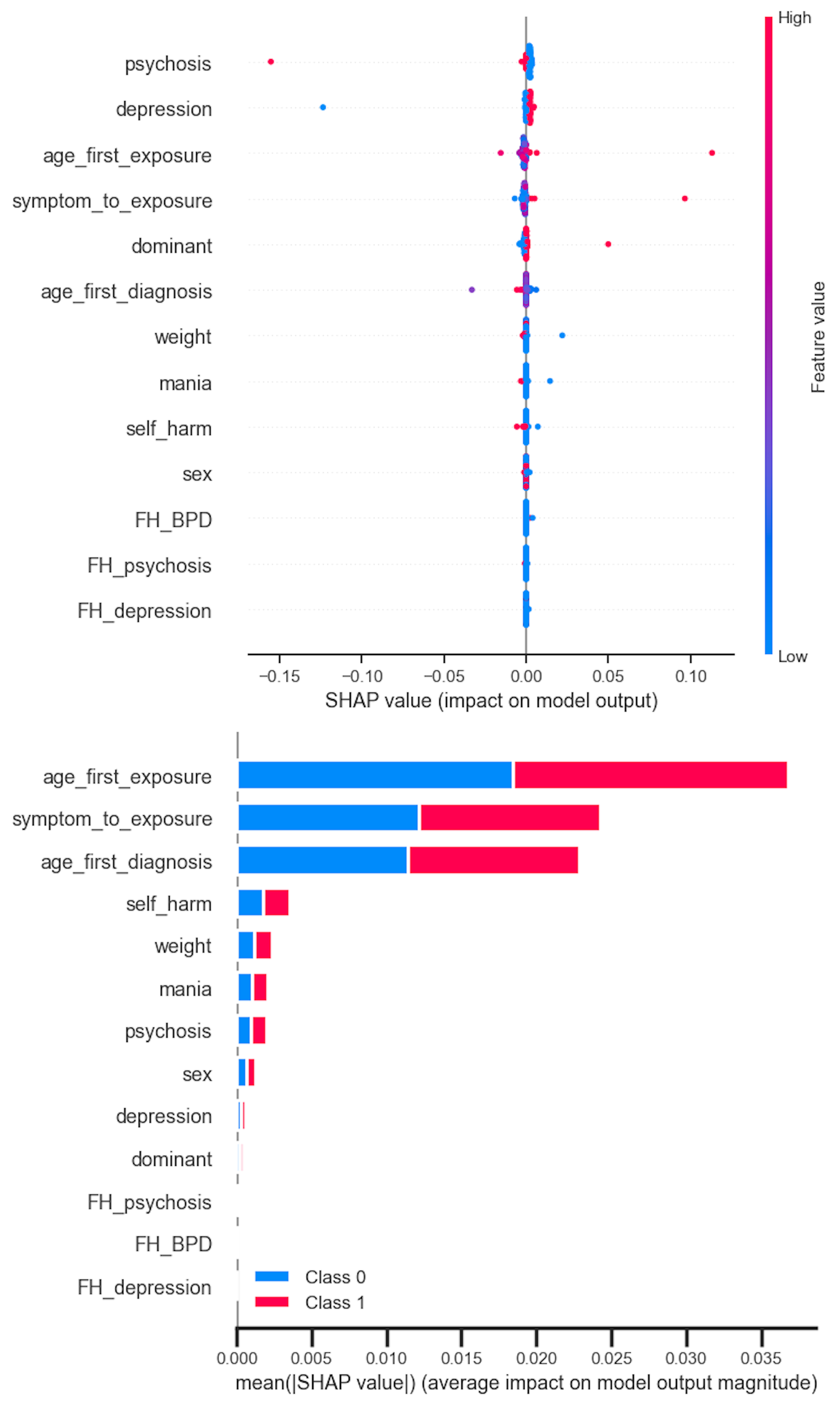

Supplement: Supplemental Information 6 [file peerj-12-17841-s006.png]
