# Supplementary material for: Predicting maintenance lithium response for bipolar disorder from electronic health records—a retrospective study
Source: PeerJ. 2024 Oct 14;12:e17841. doi: 10.7717/peerj.17841 (PMC11485101; doi:10.7717/peerj.17841)
Supplement: Supplemental Information 7 [file peerj-12-17841-s007.pdf]

NAME	DESCRIPTION
adhd	ADHD: 1= present, 0= absent before exposure
adhd_date	date of adhd diagnosis
age_first_diagnosis	Age at first recorded diagnosis
age_first_exposure	Age at exposure start
alcohol	alcohol problem: 1= present, 0= absent before exposure
alcohol_date	date of first recorded alcohol problem
anxiety	anxiety disorder: 1= present, 0= absent before exposure
anxiety_date	date of first recorded anxiety problem
asthma	asthma diagnosis
asthma_date	asthma: 1= present, 0= absent before exposure
BMI	Body mass index prior to exposure
BMI_date	date of body mass index measure
BP_date	date of blood pressure measure prior to exposure
ca	calcium blood test (before exposure start) (mmol/L)
ca_date	date of calcium blood test
cannabis	cannabis use before exposure start, 1= present, 0= absent
cannabis_date	date of first recorded cannabis use
CHD	coronary heart disease diagnosis before exposure start (including angina, MI, heart failure)
CHD_date	date of coronary heart disease diagnosis: 1= present, 0= absent
CKD3	chronic kidney disease stage 3 or more severe (eGFR<60)
cohort_end	potential end date for follow-up for individual patient
cohort_start	potential start date for follow-up for individual patient
conduct	conduct disorder diagnosis: 1= present, 2= absent
conduct_date	date of first recorded conduct disorder diagnosis
death_date	date of death
depression	depression code before exposure start: 1= present, 2= absent
depression_date	date of first recorded depression: 1= present, 0= absent
dermatitis	dermatitis code before exposure start: 1= present, 0= absent
dermatitis_date	date of first recorded dermatitis
diagnosis_date	date of first recorded bipolar disorder diagnosis
diastolic	diastolic blood pressure before exposure start
dob	date of birth (midpoint of year of birth)
dominant	dominant mood polarity: 0= unclear, 1= depression, 2= mania
eGFR_date	date of renal function blood test before exposure start
end_reason	0= end of follow-up period (31 dec 2018), 1= left practice 2= died, 3= started a mood stabiliser, 4= started an antipsychotic
ethnicity	ethnicity (broad groupings): Asian, Black, Mixed, not recorded, other, White
ethnicity_date	date ethnicity recorded (included if ever recorded)
ex_time	total exposure time (exposure_end-exposure_start)
exposure	treatment drug; 0=lithium, 1=olanzapine
exposure_end	stop date for exposure to lithium or olanzapine

exposure_start	start date for exposure to lithium or olanzapine
FH_anxiety	Family history of anxiety (ever): 1= present, 0= absent
FH_anxiety_date	date family history of anxiety recorded
FH_any	Family history of any mental health problem (ever): 1= present, 0= absent
FH_BPD	Family history of bipolar disorder (ever): 1= present, 0= absent
FH_BPD_date	date family history of bipolar recorded
FH_depression	Family history of depression (ever): 1= present, 0= absent
FH_depression_date	date family history of depression recorded
FH_LD	Family history of intellectual disability (ever): 1= present, 0= absent
FH_LD_date	date family history of intellectual disability recorded
FH_NOS	Family history of mental health problem not otherwise specified (ever): 1= present, 0= absent
FH_NOS_date	date family history of psychiatric problem NOS recorded
FH_psychosis	Family history of psychosis (ever): 1= present, 0= absent
FH_psychosis_date	date family history of psychosis recorded
FH_substance	Family history of substance misuse (ever): 1= present, 0= absent
FH_substance_date	date family history of substance misuse recorded
FH_suicide	Family history of suicide (before exposure): 1= present, 0= absent
FH_suicide_date	date family history of suicide recorded
first_date	date of first symptom of bipolar disorder
first_episode	First episode presentation: 1= depression, 2= mania
first_reg_date	date of first registration in primary care practice
HDL	High density lipoprotein (most recent pre-exposure measure - mmol/L)
HDL_date	date of most recent pre-exposure HDL measurement
hi_ca	high levels of calcium, ever before exposure (>2.65mmol/L): 1= present, 0= absent
hi_LDL	high levels of low-density lipoprotein, ever before exposure (>3mmol/L): 1= present, 0= absent
hypertension	2 measures of blood pressure >140/90 before baseline: 1= present, 0= absent
hyperthyroid	hyperthyroidism diagnosis or TSH<0.1 mU/L: 1= present, 0= absent
hyperthyroid_date	date of first hyperthyroidism
hypothyroid	hypothyroidism diagnosis or TSH>10 mU/L: 1= present, 0= absent
hypothyroid_date	date of first hypothyroidism
incident_script	first prescription for this patient: 1= likely incident prescription, 0=not incident prescription
LDL	Low density lipoprotein (most recent pre-exposure measure - mmol/L)
LDL_date	date of low-density lipoprotein measure
lo_ca	low levels of calcium, ever before exposure (<2.1mmol/L): 1= present, 0= absent
lo_HDL	low levels of high-density lipoprotein, ever before exposure (<1mmol/L): 1= present, 0= absent
mania	Mania recorded before exposure start: 1= present, 0= absent
mania_date	date of first manic episode
mania_type	type of mania experienced: 0= unclear, 1= hypomania, 2= mania, 3= mania + psychosis
migraine	migraine: 1= present, 0= absent
migraine date	date of first migraine

N_dep_b4	number of depression codes before starting lithium or olanzapine
N_man_b4	number of mania codes before starting lithium or olanzapine
OCD	obsessive compulsive disorder: 1= present, 0=absent
OCD_date	date of first obsessive compulsive disorder code
other_substance_misuse	substance misuse code (excluding cannabis); 1= present, 0= absent
other_substance_misuse_date	date of first substance misuse code (excluding cannabis)
patid	patient id (unique identifier)
PD	personality disorder: 1= present, 0= absent
PD_date	date of first personality disorder code
pracid	practice id (identifier for primary care practice)
psych_FH_date	date psychiatric family history enters (GOLD only)
psychosis	psychotic symptom code before exposure to lithium or olanzapine; 1= present, 0= absent
psychosis_date	date of first psychosis code
relationship	relationship status: 1= in a relationship, 0= not in a relationship
relationship_date	date relationship status entered
responder2	1=individual stays on exposure drug for >2 years, 0=individual stays on exposure drug for <2 years
response2_1	1=individual stays on exposure drug for >2 years, 0=individual stays on exposure drug for <1 years, .=individual stays on exposure drug for 1-2 years
self_harm	self-harm (overdose, cutting or other non-accidental injury); 1= present, 0=absent
self_harm_date	date of self-harm episode (most proximal prior to exposure start)
sex	sex; 1=male, 2=female
sleep	sleep problem: 1= present, 0=absent
sleep_date	date first presentation to GP for sleep problem
smoke_date	date smoking information captured
smoker	Smoking status at start of lithium or olanzapine treatment; 0= never smoker, 1= current smoker, 2= ex-smoker
source	Data source: 1=AURUM 2=GOLD
stress	stress: 1= present, 0= absent
stress_date	date of first presentation to GP for stress
suitable	suitable for inclusion in analysis; 1=individual has >2 years of follow-up after exposure_start, 0=individual has <2 years of follow-up after exposure_start
symptom_to_diagnosis	years from first symptom to first recorded diagnosis
symptom_to_exposure	years from first symptom to first exposure to olanzapine or lithium
systolic	value of systolic blood pressure (most proximal prior to exposure start)
T2DM	type II diabetes mellitus: 1= present, 0= absent (diagnostic code or HbA1c>48mmol/mol)
T2DM_date	date of type II diabetes mellitus diagnosis
transfer_out_date	date of exit from primary care practice
TSH	value of TSH blood test (mU/L)
TSH_date	date of TSH blood test (most proximal prior to exposure start)
weight	weight group from BMI; 0=underweight (BMI<18.5), 1=healthy weight (18.5-25), 2=overweight (25-30), 3=obese (>30)
year_exposure	year exposure started
yob	year of birth
