# Supplementary material for: Predicting maintenance lithium response for bipolar disorder from electronic health records—a retrospective study
Source: PeerJ. 2024 Oct 14;12:e17841. doi: 10.7717/peerj.17841 (PMC11485101; doi:10.7717/peerj.17841)
Supplement: Supplemental Information 8 [file peerj-12-17841-s008.pdf]

	All patients	Li >2Y
psychosis	0.327106	0.601294
depression	0.584686	0.462495
mania	0.306162	0.610635
sex	0.432688	0.530779
FH_BPD	0.106092	0.738723
FH_depression	0.108260	0.736364
FH_psychosis	0.098919	0.740540
self_harm	0.194523	0.672097
cannabis	0.102745	0.734419
anxiety	0.268067	0.611113
stress	0.142657	0.683732
sleep	0.189773	0.670088
other_substance_misuse	0.131276	0.711945
relationship	0.199050	0.690331
OCD	0.110077	0.734419
adhd	0.096688	0.742516
alcohol	0.131499	0.706812
FH_suicide	0.097899	0.744557
hi_LDL	0.141031	0.707036

lo_HDL	0.115656	0.731136
CKD3	0.111671	0.735950
T2DM	0.142625	0.729924
migraine	0.130320	0.717811
hypothyroid	0.149319	0.728139
CHD	0.128311	0.729638
FH_anxiety	0.095190	0.745354
FH_any	0.137237	0.713252
FH_LD	0.094807	0.745991
