# Supplementary material for: Predicting maintenance lithium response for bipolar disorder from electronic health records—a retrospective study
Source: PeerJ. 2024 Oct 14;12:e17841. doi: 10.7717/peerj.17841 (PMC11485101; doi:10.7717/peerj.17841)
Supplement: Supplemental Information 9 [file peerj-12-17841-s009.pdf]

Variable Name	Entropy
adhd	0.033
FH_suicide	0.028
mania	0.573
psychosis	0.591
relationship	0.390
self_harm	0.392
sex	0.679
sleep	0.379
smoker	1.066
T2DM	0.175
OCD	0.096
migraine	0.204
hypothyroid	0.201
CHD	0.146
other_substance_misuse	0.192
cannabis	0.079
alcohol	0.225
depression	0.676
FH_anxiety	0.011
FH_any	0.233
FH_BPD	0.086
FH_depression	0.097

FH_LD	0.003
FH_psychosis	0.047
anxiety	0.547
stress	0.302
hi_LDL	0.229
lo_HDL	0.119
weight	1.015
CKD3	0.094
symptom_to_exposure	8.089
dominant	0.989
age_first_exposure	9.560
age_first_diagnosis	8.743
