# Supplementary material for: Predicting maintenance lithium response for bipolar disorder from electronic health records—a retrospective study
Source: PeerJ. 2024 Oct 14;12:e17841. doi: 10.7717/peerj.17841 (PMC11485101; doi:10.7717/peerj.17841)
Supplement: Supplemental Information 10 [file peerj-12-17841-s010.pdf]

Item		Recommendation	Page No
Title and abstract	1	(*a*) Indicate the study's design with a commonly used term in the title or the abstract	1
		(*b*) Provide in the abstract an informative and balanced summary of what was done and what was found	1
Introduction			
Background/ rationale	2	Explain the scientific background and rationale for the investigation being reported	1,2
Objectives	3	State specific objectives, including any prespecified hypotheses	5-7
Methods			
Study design	4	Present key elements of study design early in the paper	2
Setting	5	Describe the setting, locations, and relevant dates, including periods of recruitment, exposure, follow-up, and data collection	2,4
Participants	6	(*a*) Give the eligibility criteria, and the sources and methods of case ascertainment and control selection. Give the rationale for the choice of cases and controls	2,4,7
		(*b*) For matched studies, give matching criteria and the number of controls per case	7
Variables	7	Clearly define all outcomes, exposures, predictors, potential confounders, and effect modifiers. Give diagnostic criteria, if applicable	5,7
Data sources/ measurement	8*	For each variable of interest, give sources of data and details of methods of assessment (measurement). Describe comparability of assessment methods if there is more than one group	7,8, Table 2, Suppl

Bias	9	Describe any efforts to address potential sources of bias	8
Study size	10	Explain how the study size was arrived at	7
Quantitative variables	11	Explain how quantitative variables were handled in the analyses. If applicable, describe which groupings were chosen and why	7, Suppl
Statistical methods	12	(*a*) Describe all statistical methods, including those used to control for confounding	2-5, Suppl
		(*b*) Describe any methods used to examine subgroups and interactions	5,8, Fig 2-3, Suppl
		(*c*) Explain how missing data were addressed	4-5
		(*d*) If applicable, explain how matching of cases and controls was addressed	Figs
		(*e*) Describe any sensitivity analyses	Table 2
Results			
Participants	13*	(a) Report numbers of individuals at each stage of study—eg numbers potentially eligible, examined for eligibility, confirmed eligible, included in the study, completing follow-up, and analysed	Table 1 2,7,8
		(b) Give reasons for non-participation at each stage	8
		(c) Consider use of a flow diagram	-
Descriptive data	14*	(a) Give characteristics of study participants (eg demographic, clinical, social) and information on exposures and potential confounders	1,4, 5,7
		(b) Indicate number of participants with missing data for each variable of interest	Zero
Outcome data	15*	Report numbers in each exposure category, or summary measures of exposure	Table 1 Suppl

Main results	16	(*a*) Give unadjusted estimates and, if applicable, confounder-adjusted estimates and their precision (eg, 95% confidence interval). Make clear which confounders were adjusted for and why they were included	Suppl
		(*b*) Report category boundaries when continuous variables were categorized	-
		(*c*) If relevant, consider translating estimates of relative risk into absolute risk for a meaningful time period	-
Other analyses	17	Report other analyses done—eg analyses of subgroups and interactions, and sensitivity analyses	Suppl
Discussion			
Key results	18	Summarise key results with reference to study objectives	7-8
Limitations	19	Discuss limitations of the study, taking into account sources of potential bias or imprecision. Discuss both direction and magnitude of any potential bias	8-9
Interpretation	20	Give a cautious overall interpretation of results considering objectives, limitations, multiplicity of analyses, results from similar studies, and other relevant evidence	7-8, Suppl
Generalisability	21	Discuss the generalisability (external validity) of the study results	8-9, Suppl
Other information			
Funding	22	Give the source of funding and the role of the funders for the present study and, if applicable, for the original study on which the present article is based	9 (with-held)
