# Supplementary material for: Predicting maintenance lithium response for bipolar disorder from electronic health records—a retrospective study
Source: PeerJ. 2024 Oct 14;12:e17841. doi: 10.7717/peerj.17841 (PMC11485101; doi:10.7717/peerj.17841)
Supplement: Supplemental Information 11 [file peerj-12-17841-s011.pdf]

Supplement—Predicting Maintenance Lithium Response for Bipolar Disorder from Electronic Health Records

Author Anonymous

ABSTRACT

Machine learning experiment details, additional figures and tables, and explanations regarding the code for the main paper are all found below.

1 MACHINE LEARNING EXPERIMENT DETAILS

In our exploratory machine learning analyses, we tested many versions of the training set, which we named following a *[X]_Y_Z* format:

Z is the label set we tried to predict:

- response (the binary response2_1 variable: less than a year exposure or more than 2)
- multi (multiclass response: less than a year exposure, between 1 and 2, or more than 2)
- exp (exposure to lithium or olanzapine)
- lithium2y (binary success with lithium: whether the patient stayed on lithium more than 2 years)

Y is the number of variables:

- 13 (informed, not listed below but exactly the same as for the 34 variables listed)
- 34 (informed, including variables not supported in the literature)

X is optional, it is a subset of the dataset:

- num (only includes numerical variables)
- cat (only categorical variables)
- bin (only binary variables)
- lit (only lithium patients)
- ola (only olanzapine patients)

Using the above legend, the following experiments were run.

```
34_resp
cat_34_resp
bin_34_resp
lit_34_resp
ola_34_resp
34_exp
cat_34_exp
bin_34_exp
34_multi
cat_34_multi
bin_34_multi
lit_34_multi
ola_34_multi
34_lithium2y
cat_34_lithium2y
bin_34_lithium2y
37_resp
cat_37_resp
bin_37_resp
lit_37_resp
ola_37_resp
37_exp
cat_37_exp
bin_37_exp
37_multi
cat_37_multi
bin_37_multi
lit_37_multi
ola_37_multi
37_lithium2y
cat_37_lithium2y
bin_37_lithium2y
13_resp
cat_13_resp
bin_13_resp
lit_13_resp
ola_13_resp
13_exp
cat_13_exp
bin_13_exp
13_multi
cat_13_multi
bin_13_multi
lit_13_multi
ola_13_multi
13_lithium2y
cat_13_lithium2y
bin_13_lithium2y
num_34_resp
num_34_exp
num_34_multi
num_34_lithium2y
num_37_resp
num_37_exp
num_37_multi
num_37_lithium2y
num_13_resp
num_13_exp
num_13_multi
num_13_lithium2y
```

2 CODE

All code developed by us is easily available. The Jupyter notebook with all of the Python 3.9 code can be found at URL WITHHELD. The repository includes an html file that can be run and inspected in any Web browser.

3 FIGURES

Figure S1

Pearson (left) pairwise linear relationships between continuous variables and Spearman (right) monotonic relationship based on rank.

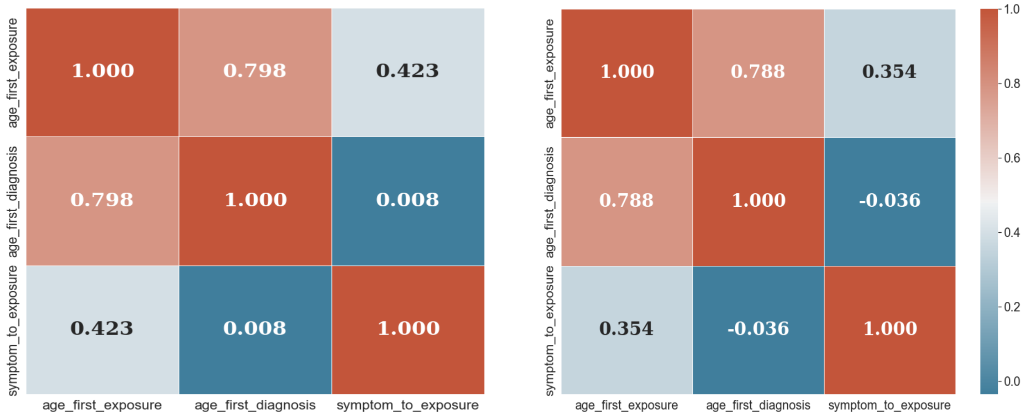

Figure S2

Chi-squared matrix for all pairs of categorical variables. Variables are ordered by importance, as informed by published research literature.

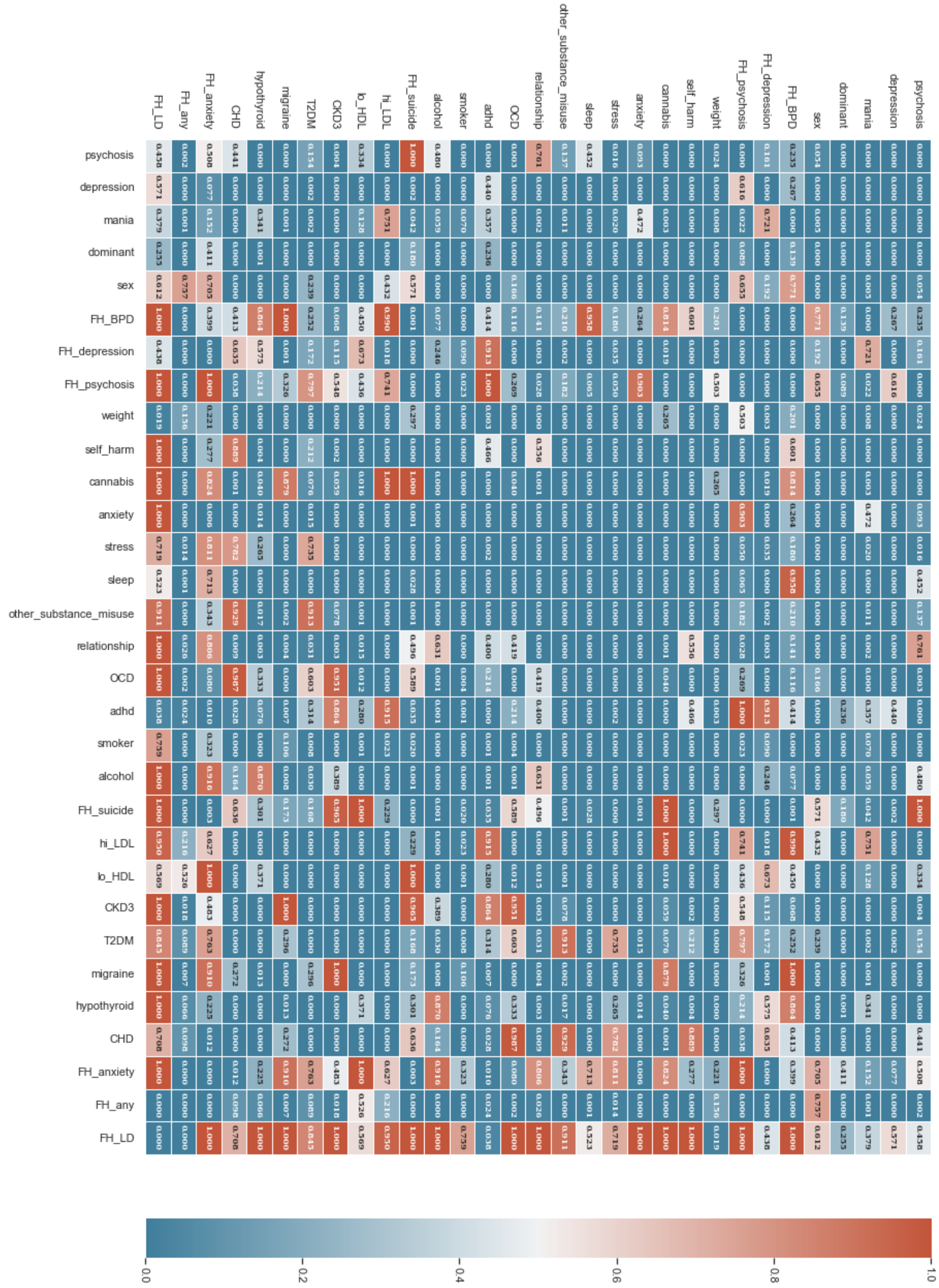

Figure S3

Distributions of the two variables most important to the primary outcome, for the 31,518 patients with more than two years of exposure. The Pearson coefficients for exposure are age_first_exposure = 0.175 and age_first_diagnosis = 0.128. Since this pair of variables has an absolute correlation coefficient of > 0.7, there is multicollinearity. Even if keeping both variables would make the use of a multiple regression model erroneous, the overlap in predictive strength is handled by the regularisation in the machine learning methods employed, and by Elastic Net via its combination of LASSO and Ridge regression.

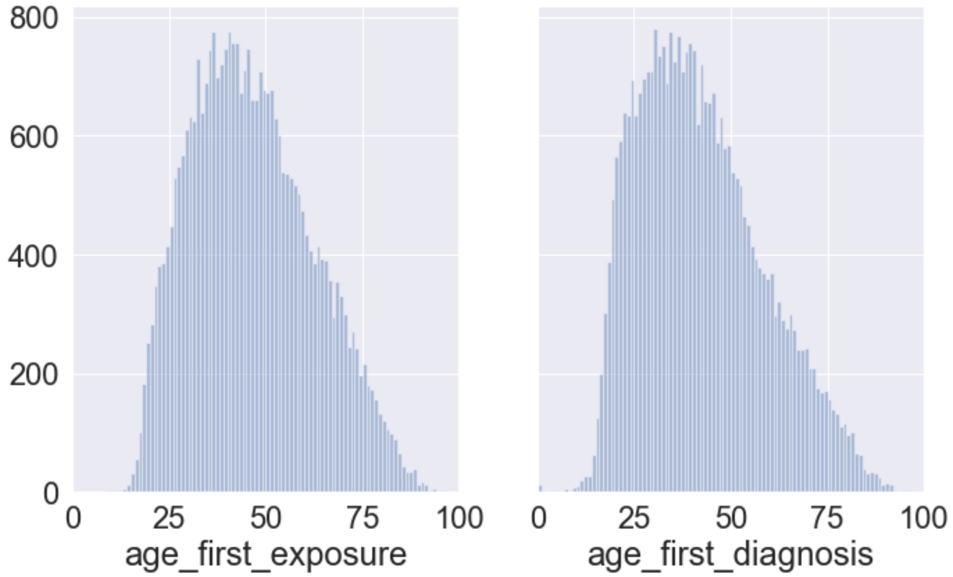

Figure S4

Plots of values of the year variable for age_first_exposure (age at exposure start), age_first_diagnosis and symptom_to_exposure (the number of years from first symptom to first exposure to Olanzapine or lithium). All outliers were manually inspected, some records removed from the data, while nonsensical values for symptom_to_exposure were set to zero to indicate that something happened at a time point unknown.

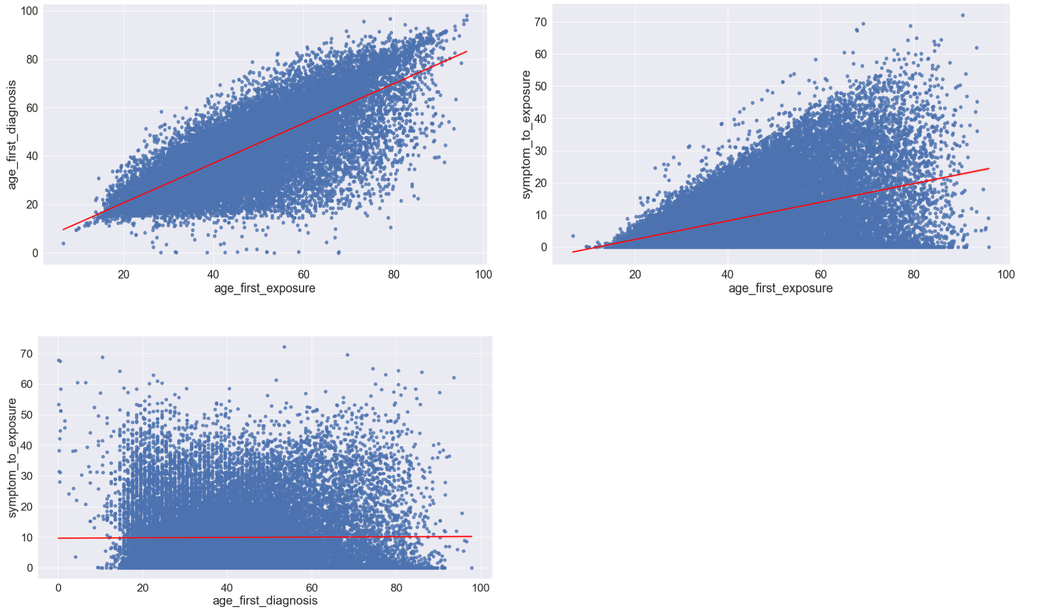

Figure S5

Age at diagnosis was established using ICD-10 criteria. Stress is a binary variable. Stress was defined using a list of non-diagnostic codes entered by the General Practitioner, indicating that the patient attended an appointment with a concern about their mental health, but no specific diagnosis was made at that time.

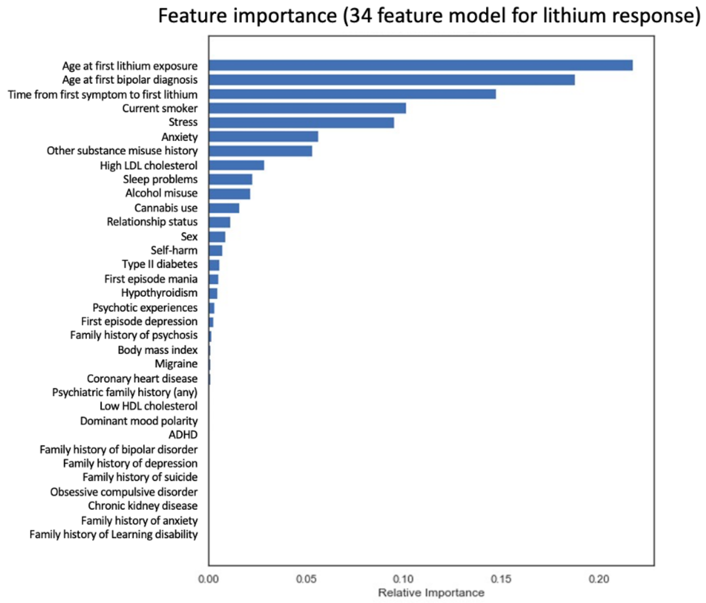

Figure S6

SHAP (Shapley additive explanations) summary statistics plot indicating the Shapley values for the 13-feature set with features in descending order of importance, when predicting if a patient is responding to lithium. High feature contribution indicated by red colour at very high impact (x-axis shows strong values for association with target), as compared to uniform expected contribution, is manifest for the two topmost features, again explaining their importance to the target value. Equally non-surprising is the similar importance of depression and dominant mood polarity. The weight feature stands out as having one high feature value contributing negatively to the target, but there are otherwise few surprises, so we abstain from further explainable AI analyses.

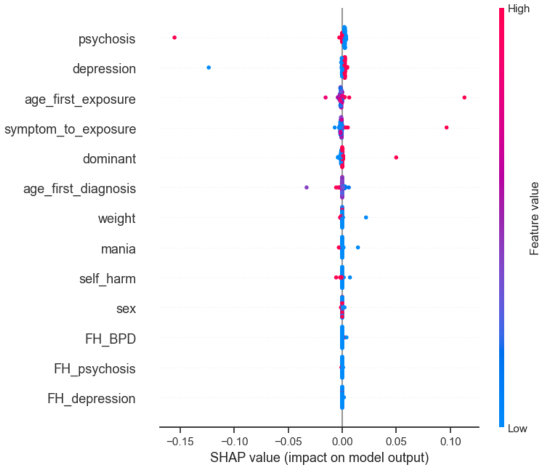

(a) Logistic Regression with Elastic Net Regularisation

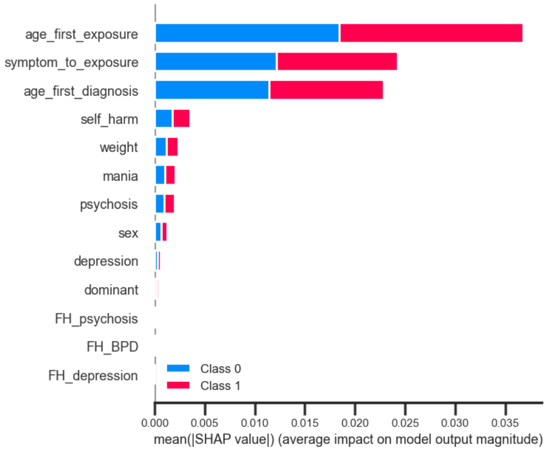

(b) Random Forest

SHAP values for models trained by different algorithms, and predicting lithium responders using the 13-feature set.

4 TABLES

Table S1

The data dictionary for the original full set of 119 variables (including the outcomes) elicited from the EHRs is listed in alphabetical order on the three pages that follow.

NAME	DESCRIPTION
adhd	ADHD: 1= present, 0= absent before exposure
adhd_date	date of adhd diagnosis
age_first_diagnosis	Age at first recorded diagnosis
age_first_exposure	Age at exposure start
alcohol	alcohol problem: 1= present, 0= absent before exposure
alcohol_date	date of first recorded alcohol problem
anxiety	anxiety disorder: 1= present, 0= absent before exposure
anxiety_date	date of first recorded anxiety problem
asthma	asthma diagnosis
asthma_date	asthma: 1= present, 0= absent before exposure
BMI	Body mass index prior to exposure
BMI_date	date of body mass index measure
BP_date	date of blood pressure measure prior to exposure
ca	calcium blood test (before exposure start) (mmol/L)
ca_date	date of calcium blood test
cannabis	cannabis use before exposure start, 1= present, 0= absent
cannabis_date	date of first recorded cannabis use
CHD	coronary heart disease diagnosis before exposure start (including angina, MI, heart failure)
CHD_date	date of coronary heart disease diagnosis: 1= present, 0= absent
CKD3	chronic kidney disease stage 3 or more severe (eGFR<60)
cohort_end	potential end date for follow-up for individual patient
cohort_start	potential start date for follow-up for individual patient
conduct	conduct disorder diagnosis: 1= present, 2= absent
conduct_date	date of first recorded conduct disorder diagnosis
death_date	date of death
depression	depression code before exposure start: 1= present, 2= absent
depression_date	date of first recorded depression: 1= present, 0= absent
dermatitis	dermatitis code before exposure start: 1= present, 0= absent
dermatitis_date	date of first recorded dermatitis
diagnosis_date	date of first recorded bipolar disorder diagnosis
diastolic	diastolic blood pressure before exposure start
dob	date of birth (midpoint of year of birth)
dominant	dominant mood polarity: 0= unclear, 1= depression, 2= mania
eGFR_date	date of renal function blood test before exposure start
end_reason	0= end of follow-up period (31 dec 2018), 1= left practice 2= died, 3= started a mood stabiliser, 4= started an antipsychotic
ethnicity	ethnicity (broad groupings): Asian, Black, Mixed, not recorded, other, White
ethnicity_date	date ethnicity recorded (included if ever recorded)
ex_time	total exposure time (exposure_end-exposure_start)
exposure	treatment drug; 0=lithium, 1=olanzapine
exposure_end	stop date for exposure to lithium or olanzapine

exposure_start	start date for exposure to lithium or olanzapine
FH_anxiety	Family history of anxiety (ever): 1= present, 0= absent
FH_anxiety_date	date family history of anxiety recorded
FH_any	Family history of any mental health problem (ever): 1= present, 0= absent
FH_BPD	Family history of bipolar disorder (ever): 1= present, 0= absent
FH_BPD_date	date family history of bipolar recorded
FH_depression	Family history of depression (ever): 1= present, 0= absent
FH_depression_date	date family history of depression recorded
FH_LD	Family history of intellectual disability (ever): 1= present, 0= absent
FH_LD_date	date family history of intellectual disability recorded
FH_NOS	Family history of mental health problem not otherwise specified (ever): 1= present, 0= absent
FH_NOS_date	date family history of psychiatric problem NOS recorded
FH_psychosis	Family history of psychosis (ever): 1= present, 0= absent
FH_psychosis_date	date family history of psychosis recorded
FH_substance	Family history of substance misuse (ever): 1= present, 0= absent
FH_substance_date	date family history of substance misuse recorded
FH_suicide	Family history of suicide (before exposure): 1= present, 0= absent
FH_suicide_date	date family history of suicide recorded
first_date	date of first symptom of bipolar disorder
first_episode	First episode presentation: 1= depression, 2= mania
first_reg_date	date of first registration in primary care practice
HDL	High density lipoprotein (most recent pre-exposure measure - mmol/L)
HDL_date	date of most recent pre-exposure HDL measurement
hi_ca	high levels of calcium, ever before exposure (>2.65mmol/L): 1= present, 0= absent
hi_LDL	high levels of low-density lipoprotein, ever before exposure (>3mmol/L): 1= present, 0= absent
hypertension	2 measures of blood pressure >140/90 before baseline: 1= present, 0= absent
hyperthyroid	hyperthyroidism diagnosis or TSH<0.1 mU/L: 1= present, 0= absent
hyperthyroid_date	date of first hyperthyroidism
hypothyroid	hypothyroidism diagnosis or TSH>10 mU/L: 1= present, 0= absent
hypothyroid_date	date of first hypothyroidism
incident_script	first prescription for this patient: 1= likely incident prescription, 0=not incident prescription
LDL	Low density lipoprotein (most recent pre-exposure measure - mmol/L)
LDL_date	date of low-density lipoprotein measure
lo_ca	low levels of calcium, ever before exposure (<2.1mmol/L): 1= present, 0= absent
lo_HDL	low levels of high-density lipoprotein, ever before exposure (<1mmol/L): 1= present, 0= absent
mania	Mania recorded before exposure start: 1= present, 0= absent
mania_date	date of first manic episode
mania_type	type of mania experienced: 0= unclear, 1= hypomania, 2= mania, 3= mania + psychosis
migraine	migraine: 1= present, 0= absent
migraine date	date of first migraine

N_dep_b4	number of depression codes before starting lithium or olanzapine
N_man_b4	number of mania codes before starting lithium or olanzapine
OCD	obsessive compulsive disorder: 1= present, 0=absent
OCD_date	date of first obsessive compulsive disorder code
other_substance_misuse	substance misuse code (excluding cannabis); 1= present, 0= absent
other_substance_misuse_date	date of first substance misuse code (excluding cannabis)
patid	patient id (unique identifier)
PD	personality disorder: 1= present, 0= absent
PD_date	date of first personality disorder code
pracid	practice id (identifier for primary care practice)
psych_FH_date	date psychiatric family history enters (GOLD only)
psychosis	psychotic symptom code before exposure to lithium or olanzapine;<br>1= present, 0= absent
psychosis_date	date of first psychosis code
relationship	relationship status: 1= in a relationship, 0= not in a relationship
relationship_date	date relationship status entered
responder2	1=individual stays on exposure drug for >2 years,<br>0=individual stays on exposure drug for <2 years
response2_1	1=individual stays on exposure drug for >2 years,<br>0=individual stays on exposure drug for <1 years,<br>.=individual stays on exposure drug for 1-2 years
self_harm	self-harm (overdose, cutting or other non-accidental injury); 1= present, 0=absent
self_harm_date	date of self-harm episode (most proximal prior to exposure start)
sex	sex; 1=male, 2=female
sleep	sleep problem: 1= present, 0=absent
sleep_date	date first presentation to GP for sleep problem
smoke_date	date smoking information captured
smoker	Smoking status at start of lithium or olanzapine treatment;<br>0= never smoker, 1= current smoker, 2= ex-smoker
source	Data source: 1=AURUM 2=GOLD
stress	stress: 1= present, 0= absent
stress_date	date of first presentation to GP for stress
suitable	suitable for inclusion in analysis;<br>1=individual has >2 years of follow-up after exposure_start,<br>0=individual has <2 years of follow-up after exposure_start
symptom_to_diagnosis	years from first symptom to first recorded diagnosis
symptom_to_exposure	years from first symptom to first exposure to olanzapine or lithium
systolic	value of systolic blood pressure (most proximal prior to exposure start)
T2DM	type II diabetes mellitus: 1= present, 0= absent (diagnostic code or HbA1c>48mmol/mol)
T2DM_date	date of type II diabetes mellitus diagnosis
transfer_out_date	date of exit from primary care practice
TSH	value of TSH blood test (mU/L)
TSH_date	date of TSH blood test (most proximal prior to exposure start)
weight	weight group from BMI; 0=underweight (BMI<18.5),<br>1=healthy weight (18.5-25), 2=overweight (25-30), 3=obese (>30)
year_exposure	year exposure started
yob	year of birth

Table S2

Binary variable correlations to the target variable (response), measured with the Simple Matching Coefficient (SMC), are listed on the following two pages. The full cohort is in the left column and the lithium maintenance responders only in the right. The symmetry of information in the compared variables made us favour SMC over Jaccard distance. No variable has a Rand similarity value of 0.75 or higher, i.e., the simple matching distance of dissimilarity is > 0.25. Except for depression, the right column has much larger similarity values, for all variables in the list.

	All patients	Li >2Y
psychosis	0.327106	0.601294
depression	0.584686	0.462495
mania	0.306162	0.610635
sex	0.432688	0.530779
FH_BPD	0.106092	0.738723
FH_depression	0.108260	0.736364
FH_psychosis	0.098919	0.740540
self_harm	0.194523	0.672097
cannabis	0.102745	0.734419
anxiety	0.268067	0.611113
stress	0.142657	0.683732
sleep	0.189773	0.670088
other_substance_misuse	0.131276	0.711945
relationship	0.199050	0.690331
OCD	0.110077	0.734419
adhd	0.096688	0.742516
alcohol	0.131499	0.706812
FH_suicide	0.097899	0.744557
hi_LDL	0.141031	0.707036

lo_HDL	0.115656	0.731136
CKD3	0.111671	0.735950
T2DM	0.142625	0.729924
migraine	0.130320	0.717811
hypothyroid	0.149319	0.728139
CHD	0.128311	0.729638
FH_anxiety	0.095190	0.745354
FH_any	0.137237	0.713252
FH_LD	0.094807	0.745991

Table S3

Conditional entropy for a curated set of variables to potentially turn into machine learning features is on the following two pages. This allows for the measuring of the potential information gain behind each variable, guiding our selection of non-redundant feature candidates: zero entropy indicates the same value for that variable for all patients, which would not assist the learning algorithm. Our preliminary feature sets were later replaced by sets in which the features represented factors supported by the research literature, which we refer to as informed rather than agnostic sets of features. One such feature set is the one of 34 variables discussed in the main paper.

Variable Name	Entropy
adhd	0.033
FH_suicide	0.028
mania	0.573
psychosis	0.591
relationship	0.390
self_harm	0.392
sex	0.679
sleep	0.379
smoker	1.066
T2DM	0.175
OCD	0.096
migraine	0.204
hypothyroid	0.201
CHD	0.146
other_substance_misuse	0.192
cannabis	0.079
alcohol	0.225
depression	0.676
FH_anxiety	0.011
FH_any	0.233
FH_BPD	0.086
FH_depression	0.097

FH_LD	0.003
FH_psychosis	0.047
anxiety	0.547
stress	0.302
hi_LDL	0.229
lo_HDL	0.119
weight	1.015
CKD3	0.094
symptom_to_exposure	8.089
dominant	0.989
age_first_exposure	9.560
age_first_diagnosis	8.743

Table S4

STROBE protocol checklist is presented on the next three pages.

Item		Recommendation	Page No
Title and abstract	1	(*a*) Indicate the study's design with a commonly used term in the title or the abstract	1
		(*b*) Provide in the abstract an informative and balanced summary of what was done and what was found	1
Introduction			
Background/ rationale	2	Explain the scientific background and rationale for the investigation being reported	1,2
Objectives	3	State specific objectives, including any prespecified hypotheses	5-7
Methods			
Study design	4	Present key elements of study design early in the paper	2
Setting	5	Describe the setting, locations, and relevant dates, including periods of recruitment, exposure, follow-up, and data collection	2,4
Participants	6	(*a*) Give the eligibility criteria, and the sources and methods of case ascertainment and control selection. Give the rationale for the choice of cases and controls	2,4,7
		(*b*) For matched studies, give matching criteria and the number of controls per case	7
Variables	7	Clearly define all outcomes, exposures, predictors, potential confounders, and effect modifiers. Give diagnostic criteria, if applicable	5,7
Data sources/ measurement	8 *	For each variable of interest, give sources of data and details of methods of assessment (measurement). Describe comparability of assessment methods if there is more than one group	7,8, Table 2, Suppl

Bias	9	Describe any efforts to address potential sources of bias	8
Study size	10	Explain how the study size was arrived at	7
Quantitative variables	11	Explain how quantitative variables were handled in the analyses. If applicable, describe which groupings were chosen and why	7, Suppl
Statistical methods	12	(*a*) Describe all statistical methods, including those used to control for confounding	2-5, Suppl
		(*b*) Describe any methods used to examine subgroups and interactions	5,8, Fig 2-3, Suppl
		(*c*) Explain how missing data were addressed	4-5
		(*d*) If applicable, explain how matching of cases and controls was addressed	Figs
		(<u>*e*</u>) Describe any sensitivity analyses	Table 2
Results			
Participants	13*	(a) Report numbers of individuals at each stage of study—eg numbers potentially eligible, examined for eligibility, confirmed eligible, included in the study, completing follow-up, and analysed	Table 1 2,7,8
		(b) Give reasons for non-participation at each stage	8
		(c) Consider use of a flow diagram	-
Descriptive data	14*	(a) Give characteristics of study participants (eg demographic, clinical, social) and information on exposures and potential confounders	1,4, 5,7
		(b) Indicate number of participants with missing data for each variable of interest	Zero
Outcome data	15*	Report numbers in each exposure category, or summary measures of exposure	Table 1 Suppl

Main results	16	(*a*) Give unadjusted estimates and, if applicable, confounder-adjusted estimates and their precision (eg, 95% confidence interval). Make clear which confounders were adjusted for and why they were included	Suppl
		(*b*) Report category boundaries when continuous variables were categorized	-
		(*c*) If relevant, consider translating estimates of relative risk into absolute risk for a meaningful time period	-
Other analyses	17	Report other analyses done—eg analyses of subgroups and interactions, and sensitivity analyses	Suppl
Discussion			
Key results	18	Summarise key results with reference to study objectives	7-8
Limitations	19	Discuss limitations of the study, taking into account sources of potential bias or imprecision. Discuss both direction and magnitude of any potential bias	8-9
Interpretation	20	Give a cautious overall interpretation of results considering objectives, limitations, multiplicity of analyses, results from similar studies, and other relevant evidence	7-8, Suppl
Generalisability	21	Discuss the generalisability (external validity) of the study results	8-9, Suppl
Other information			
Funding	22	Give the source of funding and the role of the funders for the present study and, if applicable, for the original study on which the present article is based	9 (with-held)
