# Supplementary material for: Predicting maintenance lithium response for bipolar disorder from electronic health records—a retrospective study
Source: PeerJ. 2024 Oct 14;12:e17841. doi: 10.7717/peerj.17841 (PMC11485101; doi:10.7717/peerj.17841)
Supplement: Supplemental Information 2 [file peerj-12-17841-s002.pdf]

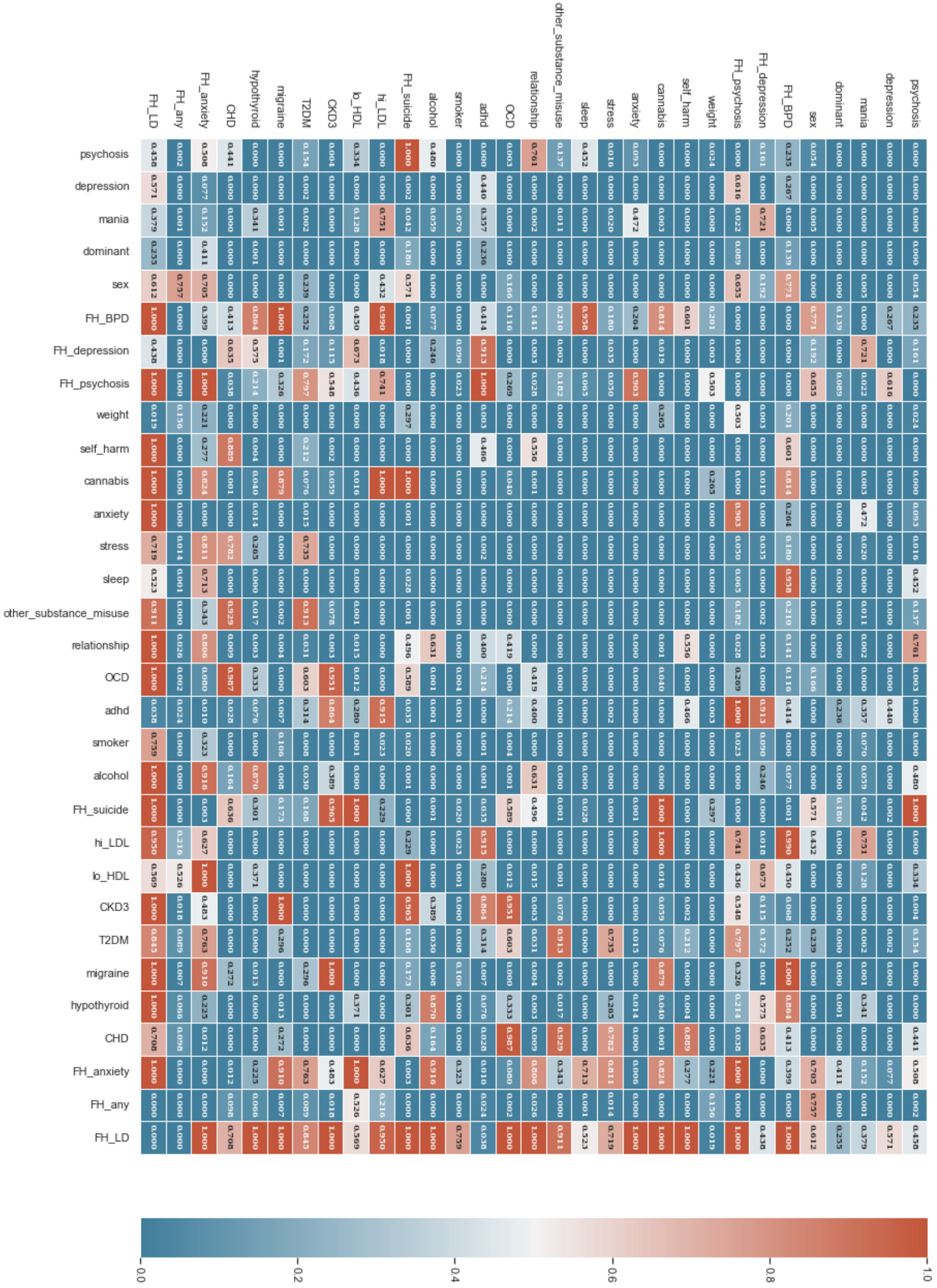

psychosis
depression
mania
dominant
sex
FH_BPD
FH_depression
FH_psychosis
weight
self_harm
cannabis
anxiety
stress
sleep
other_substance_misuse
relationship
OCD
adhd
smoker
alcohol
FH_suicide
hi_LDL
lo_HDL
CKD3
T2DM
migraine
hypothyroid
CHD
FH_anxiety
FH_any
FH_LD
0.0
0.2
0.4
0.6
0.8
1.0
